# Supplementary material for: An Overcomplete Approach to Fitting Drift-Diffusion Decision Models to Trial-By-Trial Data
Source: Front Artif Intell. 2021 Apr 9;4:531316. doi: 10.3389/frai.2021.531316 (PMC8064018; doi:10.3389/frai.2021.531316)
Supplement: Supplementary file 1 [file DataSheet1.docx]

**Supplementary Information**

**Appendix 1 : Summary of the variational Laplace approach**

To begin with, note that the mathematical form of all generative models $m$ that we consider here can be written as follows (for notational convenience, we ignore the dependence on decision outcomes):

(A1)

where $y$ are the fitted data (typically: RTs or RT moments), are model parameters, $\varepsilon$ are model residuals, and $g$ is a mapping that depends upon the generative model. For the overcomplete approach, the observation mapping $g$ is given by the self-consistency equation, having replaced native DDM parameters with their corresponding dummy variables (cf. sections 3.a and 3.b of the main text). The observation mapping of other approaches are described in Appendix 2 and 3 below.

Recall that the variational Laplace scheme is an iterative algorithm that indirectly optimizes an approximation to both the model evidence $p\left(y\left|m\right.\right)$ and the posterior density . The key trick is to decompose the log model evidence into:

, (A2)

where is any arbitrary density over the model parameters, $D_{KL}$ is the Kullback-Leibler divergence and the so-called *free energy* $F\left(q\right)$ is defined as:

, (A3)

where $S\left(q\right)$ is the Shannon entropy of $q$ and the expectation is taken under $q$.

From equation A2, maximizing the functional $F\left(q\right)$ w.r.t. $q$ indirectly minimizes the Kullback-Leibler divergence between and the exact posterior . This decomposition is complete in the sense that if , then $F\left(q\right)=\ln p\left(y\left|m\right.\right)$.

The variational Laplace algorithm iteratively maximizes the free energy $F\left(q\right)$ under simplifying assumptions (see below) about the functional form of $q$, rendering $q$ an approximate posterior density over model parameters and $F\left(q\right)$ an approximate log model evidence (Daunizeau, 2017; Friston et al., 2007). The free energy optimization is then made with respect to the sufficient statistics of $q$, which makes the algortithm generic, quick and efficient.

Under normal i.i.d. model residuals, the likelihood function writes:

(A4)

where $\lambda$ is the residuals' precision or inverse variance hyperparameter.

We also use Gaussian priors for model parameters and gamma priors for precision hyperparameters $p\left(\lambda\left|m\right.\right)=Ga\left(\varpi_{0},\kappa_{0}\right)$.

In what follows, we derive the variational Laplace algorithm under a "mean-field" separability assumption between parameters and hyperparameters, i.e.: . We will see that this eventually yields a Gaussian posterior density on model parameters, and a Gamma posterior density $q\left(\lambda\right)=Ga\left(\varpi,\kappa\right)$ on the precision hyperparameter.

First, let us note that, under the Laplace approximation, the free energy bound on the log-model evidence can be written as:

(A5)

where is the number of parameters, is the gamma function, is the digamma function, and is defined as:

(A6)

Given the Gamma posterior $q\left(\lambda\right)$ on the precision hyperparameter, can be simply expessed as follows:

(A7)

where we have ignored the terms that do not depend upon , and $\left\langle\lambda\right\rangle=E\left[\lambda\left|y,m\right.\right]=\varpi/\kappa$ is the posterior mean of the data precision hyperparameter $\lambda$.

The variational Laplace update rule for the approximate posterior density on model parameters now simply reduces to an update rule for its sufficient statistics:

(A8)

In Equation A8, the first-order moment $\mu$ of is obtained from the following Gauss-Newton iterative gradient ascent scheme:

(A9)

where the gradient and Hessians of are given by:

(A10)

At convergence of the above gradient ascent, the approximate posterior density on the precision hyperparameter is updated as follows:

(A11)

where $n_{y}$ is the number of data samples.

The variational Laplace scheme alternates between Equations A8 and A11 iteratively until convergence of the free energy.

At this point, we note that, for all estimation methods, we set the prior probability density functions as follows:

- , i.e. the prior mean of model parameters is $\mu_{0}=0$ and their prior variance is $\Sigma_{0}=I$. For group studies, this prior setting can be replaced with parametric empirical Bayes priors (Daunizeau, 2019).
- $p\left(\lambda\left|m\right.\right)=Ga\left(1,\kappa_{0}\right)$, where $\kappa_{0}=V\left[y\right]$ is the variance of the dependent variable $y$. In other words, the data is *a priori* assumed to be driven entirely by noise. This ensures that the prior and likelihood components of are balanced when the variational Laplace algorithm starts.

This completes the description of the variational Laplace approach.

For more details, we refer the interested reader to the existing literature on variational approaches to approximate bayesian inference (Beal, 2003; Daunizeau, 2017; Friston et al., 2007). We note that the above variational Laplace approach is implemented in the academic opensource VBA toolbox (Daunizeau et al., 2014): <https://mbb-team.github.io/VBA-toolbox/>.

**Appendix 2: Method of moments**

Let $p\left(\tau\left|o,v,x_{0},b,\sigma\right.\right)$ be the theoretical probability density function of hitting times $\tau$, conditional on the decision outcome $o$ and DDM parameters. Here, we evaluate $p\left(\tau\left|o,v,x_{0},b,\sigma\right.\right)$ using a fast and accurate semi-analytical approach which was derived for the "vanilla" DDM case (Navarro and Fuss, 2009). Although not generalizable to more complex DDM variants, this eschews the need for sampling scheme or numerical solvers of the underlying Fokker-Planck equation, which are computationally very demanding.

Then, RT conditional (central) moments $E\left[\left(\tau-\bar{\tau}\right)^{k}\left|o,v,x_{0},b,\sigma\right.\right]$ are simply given by:

$E\left[\left(\tau-\bar{\tau}\right)^{k}\left|o,v,x_{0},b,\sigma\right.\right]=\int\left(\tau-\bar{\tau}\right)^{k}p\left(\tau\left|o,v,x_{0},b,\sigma\right.\right)d\tau\begin{matrix} \\ \end{matrix}$ (A12)

where $k$ is the moment's order (e.g., $k=2$: variance, $k=3$: skewness), $\bar{\tau}=E\left[\tau\left|o,v,x_{0},b,\sigma\right.\right]$ is the conditional mean response time. We evaluate the right-hand term of Equation A12 using numerical integration, on a predefined integration grid.

The method of moments then attempts to match the empirical and theoretical moments of RT data (conditional on decision outcomes). This is formalized through a generative model of the form given in Equation A1 above, where $y$ is the set of empirical RT moments (here, up to order 3) and the observation mapping is made of the corresponding set of theoretical moments (cf. Equation A12). The variational Laplace approach then yields an approximate posterior density on DDM parameters, under the assumptions given in Appendix 1. This completes the summary description of the method of moments.

The method of moments is known to be fast and robust, which is why it is an established approach for inference on population parameters in the statistical literature (Newey and West, 1987). In the context of DDM applications (Grasman et al., 2009; Pedersen and Frank, 2020; Vandekerckhove and Tuerlinckx, 2008; Voss and Voss, 2007; Wagenmakers et al., 2007, 2008; Wiecki et al., 2013), it lumps all trial-by-trial variations into statistical moments of the RT distribution. Importantly, this may induce some severe information loss in some contexts.

For example, let us consider experimental designs where DDM drift rates are varied systematically over trials. The method of moments would account for such variations by marginalizing the conditional RT distribution over the drift rate trial-by-trial distribution:

$\begin{matrix} p\left(\tau\left|o,x_{0},x*,\sigma\right.\right)=\int p\left(\tau\left|o,v,x_{0},b,\sigma\right.\right)p\left(v\right)dv\begin{matrix} \\ \end{matrix} \\ \approx\frac{1}{n}\sum_{i=1}^{n} p\left(\tau\left|o,v_{i},x_{0},b,\sigma\right.\right) \end{matrix}$ (A13)

where $n$ is the number of trials, $v_{i}$ is the predicted drift rate at trial $i$, and $p\left(\tau\left|o,v_{i},x_{0},b,\sigma\right.\right)$ is the corresponding RT distribution. One can see that the resulting marginal distribution $p\left(\tau\left|o,x_{0},b,\sigma\right.\right)$ will be blind to many forms of predictable trial-by-trial drift rate variations. To begin with, $p\left(\tau\left|o,x_{0},b,\sigma\right.\right)$ is invariant to any arbitrary permutation of the trials. In addition, as we will see below, increasing drift rate variability has almost the same effect on the marginal distribution $p\left(\tau\left|o,x_{0},b,\sigma\right.\right)$ than increasing the variance $\sigma^{2}$ of neural stochastic perturbation terms. This is summarized on Figure A1 below.

**Figure A1: impact of the trial-by-trial variance of drift rate.** Same format as Fig 1 of the main text. Note that the mean drift rate $\bar{v}$ was fixed to its default value, i.e.: $\bar{v}=1/2$, which corresponds to black dotted lines on all panels of Figures 1 to 4).

As one can see, the trial-by-trial variance of drift rate essentially has no impact on the marginal distribution $p\left(\tau\left|o,x_{0},b,\sigma\right.\right)$ as long as it is below or of the same order of magnitude than the mean drift rate. From this point onwards, it decreases the hitting time's mean and its variance, increases its skewness, and increases the entropy of the outcome probability. This effect is qualitatively identical to the impact of the noise's standard deviation $\sigma$ (cf. Figure 3 of the main text). Practically speaking, Figure A1 implies that the method of moments may confuse trial-by-trial variations of drift rates with the effect of stochastic perturbations. In retrospect, this is expected, because any form of trial-by-trial drift-rate variations will eventually conspire with stochastic perturbations to inflate trial-by-trial RT variations. We note that this argument generalizes to trial-by-trial variations in other model parameters.

**Appendix 3: Method of the trial means**

A possible improvement over the above method of moments is to match trial-by-trial RT data to their corresponding theoretical mean. This is typically done by evaluating trial-specific likelihood functions from samplers of the DDM model or related numerical approaches, which may then be used within a probabilistic framework for parameter estimation (Fontanesi et al., 2019a, 2019b; Gluth and Meiran, 2019; Moens and Zenon, 2017; Pedersen et al., 2017; Wabersich and Vandekerckhove, 2014). For numerical expediency, we here rather rely on the variational Laplace described in Appendix 1. In brief, the method of trial means relies upon a generative model of the form given in Equation A1, where $y$ is now the raw RT data trial series and the observation mapping is made of the corresponding set of trial-by-trial expected RTs (conditional on decision outcomes). The latter are derived from the following analytical expression for the RT conditional mean, which is valid under the "vanilla" DDM (Srivastava et al., 2016):

$E\left[\tau\left|o\right.,v,x_{0},b,\sigma\right]=\frac{b}{v}\left(2\coth\left(\frac{2v b}{\sigma^{2}}\right)-\left(1+o\frac{x_{0}}{b}\right)\coth\left(\left(1+o\frac{x_{0}}{b}\right)\frac{v b}{\sigma^{2}}\right)\right)$ (A14)

where $o\in\left\{-1,1\right\}$ is the decision outcome. One can then evaluate Equation A14 for each trial, given its corresponding set of DDM parameters. In particular, if one knows how drift rates vary over trials, then one can predict the ensuing expected RT variations. The variational Laplace treatment of the ensuing generative model then yields an approximate posterior density on the remaining DDM parameters. This completes the summary description of the method of trial means.

**Appendix 4: parameter recovery analysis**

Parameter recovery analyses aim at evaluating how accurate parameter estimates are. This is done by comparing simulated and estimated parameters using Monte-Carlo simulation series, which we now detail.

Let $\theta=\left\{v,x_{0},b,\sigma,T_{ND}\right\}$ be the set of all DDM parameters. In what follows, we refer to $\theta_{ij}$ as *i*^th^ element of $\theta$ at the *j*^th^ Monte-Carlo simulation. At each Monte-Carlo simulation, the vector of simulated parameters is randomly drawn from a standard normal distribution (passed through its corresponding mapping, see section 3.b of the main text). Given , we then simulate a series of N=200 DDM trials according to Equation 2. The ensuing trial series of choice outcomes and response times is then fed to each parameter estimation method, each of which outputs a vector of estimated parameters, where *k* is the estimation method’s index. We repeat this procedure $N_{mc}=1000$ times, yielding a series of 1000 simulated parameter sets, and their corresponding 1000 estimated parameters sets (per estimation method). Should , then parameter recovery would be perfect. The accuracy of parameter recovery can thus be visually evaluated when plotting estimated parameter against simulated parameters (cf. Figures 7, 9, 11 and13).

To further quantifying the accuracy of parameter recovery, we measure the mean relative estimation error $REE_{j}^{(k)}$ for each Monte-Carlo simulation and each estimation method:

$REE_{j}^{(k)}=\frac{1}{n_{\theta}}\sum_{i=1}^{n_{\theta}} \left|\frac{\theta_{ij}-\hat{\theta}_{ij}^{(k)}}{\theta_{ij}}\right|$ (A15)

where $n_{\theta}$ is the number of DDM parameters to be estimated (see main text). Between-method comparisons can then be compared using pairwise statistical tests. We then use one-sample F-tests on the difference $\Delta\log\left(REE\right)=\sum_{j=1}^{N_{mc}} \left(\log REE_{j}^{(k)}-\log REE_{j}^{(k')}\right)$ of log-transformed estimation errors. Log-transformed estimation errors are summarized on Figure 15 of the main text.

We also quantify pairwise non-identifiability issues, which arise when the estimation method confuses two parameters with each other. We do this using so-called “recovery matrices”, which summarize whether variations (across the 1000 Monte-Carlo simulations) in estimated parameters faithfully capture variations in simulated parameters. We first z-score simulated and estimated parameters across Monte-Carlo simulations. We then regress each estimated parameter against all simulated parameters through the following multiple linear regression model:

$\hat{\theta}_{ij}^{(k)}=\sum_{i'=1}^{n_{\theta}} \beta_{ii'}^{(k)}\theta_{i'j}+\varepsilon_{ij}^{(k)}$ (A16)

where $\beta_{ii'}^{(k)}$ are regression weights, and $\varepsilon_{ij}^{(k)}$ are regression residuals. Here, regression weights are partial correlation coefficients between simulated and estimated parameters (across Monte-Carlo simulations). More precisely, $\beta_{ii'}^{(k)}$ quantifies the impact that variations of the simulated parameter have on variations of the estimated parameter , conditional on all other simulated parameters. Would parameters be perfectly identifiable, then $\beta_{ii}^{(k)}\approx1$ and $\beta_{ii'}^{(k)}\approx0 \forall i'\neq i$. Recovery matrices simply report the squared regression weights for each simulated parameter (cf. Figure 8, 10, 12 and 14 of the main text).

Note that the regression model in Equation A16 effectively decomposes the observed variability in the estimated parameter into “correct variations” that are induced by variations in the corresponding simulated parameter , and “incorrect variations” that are induced by the remaining simulated parameters (with $i'\neq i$). In turn, this enables the derivation of the following identifiability index $\Delta V^{(k)}$:

(A17)

where the first and the second summands of the right-hand term of Equation A17 are the amount of “correct” and “incorrect” variations, respectively. The identifiability index is maximal when the amount of “correct variations” dominates the amount of “incorrect variations”. Mean identifiability indices are summarized on Figure 15 of the main text.

**Appendix 5: evaluating the computational optimality of a DDM**

Recall that the DDM has been shown to be the optimal solution to the computational problem of trading speed with accuracy when making online value-based decisions (Tajima et al., 2016). This speed-accuracy tradeoff arises because the decision system needs to accumulate noisy value signals before making a reliable decision. Arguably, the decision system cannot control the upstream decision variables, such as the evidence strength (i.e. the drift rate $v$), or the reliability of value signals (i.e. the noise's standard deviation $\sigma$). However, it can set the bound's height $b$ so as to optimize the ensuing speed-accuracy tradeoff. More precisely, the system can favor decision accuracy by increasing $b$, or improve decision speed by decreasing $b$. In context of perceptual decision making, one can score the efficiency of any $b$ setting, in terms of the ensuing reward rate $RR\left(b\right)$ (Balci et al., 2011; Bogacz et al., 2006; Zhang, 2012):

$RR\left(b\right)=\frac{P_{c}\left(b\right)}{E\left[HT\left|b\right.\right]}$ (A18)

where $E\left[HT\left|b\right.\right]$ is the expected hitting time, $P_{c}\left(b\right)\triangleq P\left(o=correct\left|b\right.\right)$ is the ensuing probability of making a correct decision, and we have neglected non-decision times and inter-trial intervals. The “optimal” bound's height is such that it maximizes the reward rate. Now the system may not have set the bound's height to its optimal value. The optimality of the system can thus be measured in terms of the ratio between the actual efficiency of the system and its optimal efficiency:

(A19)

where $\hat{b}$ is the estimated bound's height of a given participant. Equation A19 can be extended to situations where drift rate changes in a trial-by-trial fashion, by averaging the optimality score over trials. This makes the average optimality score a measure of how well the system adapts to the global statistics of decisions it has to make.

Figure A2 below shows a typical example of the derivation of the optimality score, based upon the estimated DDM parameters of a study participant (and the corresponding sequence of drift rates over trials) under the default/alternative frame of reference.

The optimality score of this participant is estimated as . In other terms, this participant's decisions seem to arise from a DDM decision system that is close to its optimal setting, in terms of the underlying speed-accuracy tradeoff.

| 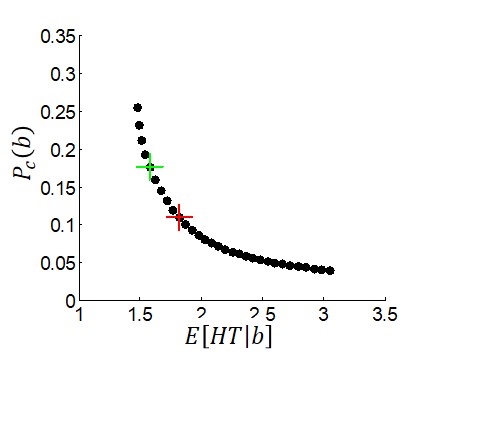 | 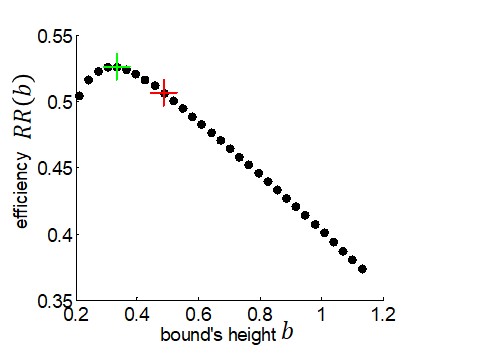 |
| --- | --- |
| **Figure A2: Model-based evaluation of DDM optimality**. **Left panel**: The probability $P_{c}\left( b \right)$ of making a correct decision (y-axis) is plotted against the expected decision time $E\left[ HT\left\vert b \right. \right]$ (y-axis) for each possible bound height. The green and red crosses depict the optimal and actual speed-accuracy tradeoffs, respectively. **Right panel**: The efficiency $e\left( b \right)$ (y-axis) is plotted for each possible bound height *b*. The green cross depicts the optimal efficiency and the red cross depicts the actual efficiency achieved for this participant. | |

**Appendix 6: The "ballistic" limit of drift rates**

In this work, we assert that drift rates are bounded above and below (cf. section 3.b if the main text). More precisely, we enforce the following constraint on drift rate estimators:

$\left|v\right|\leq\frac{b+\left|x_{0}\right|}{\min\left(RT\right)-T_{ND}}$ (A20)

where the minimum RT is taken over trials. In what follows, we refer to the right-hand term of Equation A20 as the "ballistic" limit of drift rates.

So-called “linear ballistic accumulation models” can be understood as non-stochastic variants of the DDM, whereby evidence accumulation follows a straight line with a slope equal to the DDM's drift rate (Goldfarb et al., 2014; Osth et al., 2017). Under such ballistic models, it is not unreasonable to interpret RT variability in terms of drift rate variability. In turn, drift rates would necessarily obey Equation A20.

Intuitively, this result generalizes to DDMs because the net effect of adding stochastic perturbations to ballistic evidence accumulation is to accelerate decision times. Consider the vanilla DDM, where the drift rate is fixed but unknown. If there is no decision error, then the RT distribution is approximately centred around the corresponding "ballistic" RT. In turn, a "ballistic" drift rate estimate based upon the minimum RT would necessarily be greater than the true drift rate, hence the "ballistic limit". This reasoning is also valid for decision errors, though there is no trivial ballistic equivalent to decision errors.

To check this reasoning, we performed a series of 2000 Monte-Carlo simulations, where we varied randomly all DDM parameters (without imposing the "ballistic limit" to drift rates). Figure A3 below compares the (unconstrained, randomly drawn) drift rates to their theoretical "ballistic limit".

|  |
| --- |
| **Figure A3: Validity of the "ballistic limit" of drift rates**. The right-hand side of Equation A20 (ballistic limit, y-axis) is plotted against the left-hand-side of Equation A20 (absolute value of unconstrained drift rate, x-axis). Each dot is a Monte-Carlo simulation of 200 trials of a DDM with randomly drawn parameters. The red line indices the identity mapping, i.e. equality between drift rates and their ballistic limit. |

One can see that the ballistic limit is always greater than unconstrained drift rates, irrespective of the actual DDM parameter setting. This validates the “ballistic” limit of drift rates.

**Appendix 7: Prior preferences: alternative analysis results**

In the main text, we report the results of value-based decision data analysis using the overcomplete approach. Here, we summarize the results of analyses of the same dataset, this time using the method of moments and the method of trial means. Note that we used the exact same statistical testing procedures in all cases.

To begin with, we tested the group-level significance of estimated initial conditions $\hat{x}_{0}$. For all estimation methods, it is significant for the default frame (method of moments: F=386.2, dof=[1,23], p<10^-4^, method of trial means: F=60.1, dof=[1,23], p<10^-4^) but not for the native frame (method of moments: F=0.81, dof=[1,23], p=0.38, method of trial means: F=1.82, dof=[1,23], p=0.19). This result is qualitatively similar to the overcomplete approach.

We then asked whether inter-individual differences in $\hat{x}_{0}$ predict inter-individual differences in observed choice and RT biases. Figure A4 and A5 below summarize these relationships for method of moments and the method of trial means, respectively.

|  |
| --- |
| **Figure A4: Model-based analyses of choice and RT data: method of moments**. Same format as Figure 17. |

When estimated with the method of moments, inter-individual differences in $\hat{x}_{0}$ significantly correlates with choice bias (r=0.51, p=0.01) but not with RT bias (r=0.34, p=0.11).

|  |
| --- |
| **Figure A5: Model-based analyses of choice and RT data: method of trial means**. Same format as Figure 17. |

When estimated with the method of trial means, inter-individual differences in $\hat{x}_{0}$ significantly correlates with RT bias (r=0.70, p=0.0002) but not with choice bias (r=0.34, p=0.11).

Taken together, these results imply that the overcomplete approach provides estimates of inter-individual differences that more reliably relate to observable biases than the method of moments or the method of the trial means.

Lastly, we note that that, using the overcomplete approach, analyzing the data under a generalized variant of the DDM (with, e.g., collapsing bounds) yields qualitatively similar results as under the vanilla DDM. However, the bound's exponential decay rate was not significant at the group-level (F=0.89, dof=[1,23], p=0.35). In other terms, there is no strong evidence for exponentially decaying bounds in this dataset, which is why we report the results of the vanilla DDM in the main text.
